# Post-stroke seizures in animal models: a systematic review and meta-analysis

**DOI:** 10.3389/fnins.2025.1716816

**Published:** 2025-12-02

**Authors:** Kayla D. L. Csernyanszki, Nmazule K. Nyenke-Wofuru, McKenzee M. Olsen, Amelie V. Grenier, Hazel Hwata, Ana C. Klahr

**Affiliations:** 1 Department of Social Sciences, Augustana Faculty, University of Alberta, Camrose, AB, Canada; 2 Department of Psychology, University of Alberta, Edmonton, AB, Canada; 3 Centre for Neuroscience, University of Alberta, Edmonton, AB, Canada

**Keywords:** post-stroke seizures, ischemic stroke, intracerebral hemorrhage, animal models, lesion volume, neurological deficits

## Abstract

**Background:**

Post-stroke seizures (PSS) are a common complication of stroke and can exacerbate neurological injury, yet their study in preclinical models remains limited. Understanding the relationship between PSS and outcomes in animal models is critical for improving translational research and informing therapeutic strategies.

**Objective:**

To systematically review and meta-analyze the incidence, consequences, and methodological quality of studies investigating PSS in animal models of ischemic stroke (IS) and intracerebral hemorrhage (ICH).

**Methods:**

A systematic search of Embase, Medline, Scopus, and Web of Science (June 2024, updated May 2025) identified original, peer-reviewed animal studies published after 1999 that reported seizures and outcomes (lesion volume, neurological deficit scores, behavior, edema, inflammation) without interventional treatments. Data extraction, risk-of-bias assessment, and a random-effects meta-analysis was performed for lesion volume.

**Results:**

Of 6,005 studies screened, 10 met inclusion criteria, with eight eligible for meta-analysis. Seizure incidence ranged from 17.5–82% in focal ischemia and 45–67% in ICH models. Lesion volume was the most commonly measured outcome. Meta-analysis revealed that seizures were associated with larger lesion volumes in focal ischemia models (Hedge’s G = 1.598, *p* = 0.038) but not in ICH models (Hedge’s G = 0.180, *p* = 0.468). Across studies, seizures were linked to more severe neurological deficits in focal ischemia but showed no consistent effect in ICH. Risk-of-bias assessment indicated high risk in all studies, with frequent methodological limitations including lack of random outcome assessment, use of only young male animals, and absence of *a priori* sample size calculations. Publication bias was suggested by funnel plot asymmetry.

**Conclusion:**

This review highlights a scarcity of rigorous preclinical studies on PSS, substantial heterogeneity across animal models, and methodological limitations that hinder translatability. Findings suggest a differential impact of stroke type on seizure outcomes, with focal ischemia-associated seizures linked to larger lesions and poorer neurological function. Future research should employ long-term, rigorously designed studies using diverse animal populations, standardized seizure monitoring, and careful reporting to enhance clinical relevance and guide therapeutic development.

## 1 Introduction

Globally, stroke is a critical public health concern, with an estimated one in four individuals experiencing a stroke during their lifetime (Feigin et al., 2025). It represents the second leading cause of death and disability and accounts for one-third of new-onset epilepsy in the elderly population (Feigin et al., 2025; He et al., 2024). This is a notable concern for stroke survivors as post-stroke seizures (PSS) can exacerbate secondary injury, extend hospital stays, increase mortality, and worsen long-term patient outcomes (Xu, 2019; Tanaka et al., 2024).

The reported incidence of PSS varies widely due to differences in study populations and definitions (Xu, 2019; Tanaka et al., 2024; Camilo and Goldstein, 2004; Freiman et al., 2024), while post-stroke epilepsy (PSE) occurs in 2–14% in ischemic stroke (IS) patients and 10–20% of individuals with an intracerebral hemorrhage (ICH) (Tanaka et al., 2024; Camilo and Goldstein, 2004; Ryu et al., 2024; Myint et al., 2006). Clinically, PSS are common (Ryu et al., 2024; Nandan et al., 2023), yet they are rarely considered as a therapeutic endpoint in preclinical stroke studies. Animal studies offer a distinct advantage by allowing for the control of variables (e.g., sex, stroke size, comorbidities) that contribute to the heterogeneity observed in patient data. This controlled approach facilitates the elucidation of precise relationships between pathophysiological mechanisms and stroke outcomes. Additionally, it allows for investigation of prophylactic administration of anticonvulsants, for which current clinical outcomes yield mixed results (Tanaka et al., 2024). However, despite PSS representing a clinical concern and a contributor to stroke pathophysiology, the relationship between PSS and outcomes in animal models remains under-investigated.

This deficiency is critical, as high-quality preclinical studies investigating the relationship between the incidence, lesion volume, and functional and neurological outcomes of PSS are scarce. Even fewer clinical randomized control trials test the efficacy of antiepileptic drugs (AED) treatments (Leo et al., 2020; Brigo et al., 2018). As the incidence of stroke and, therefore, PSS and PSE escalate (Feigin et al., 2025; Cheng et al., 2024), so too does the demand for better identification, risk assessment, and treatment to prevent the potentially adverse side effects of seizures through prevention and limitation of their development following stroke. The scarcity of studies on PSS and PSE in animal models creates a gap in the literature crucial to enhancing researcher and clinician knowledge and study design. Other reviews of the preclinical stroke literature focus on AED and neuroprotective drug efficacy, animal model and study quality, and epileptogenesis (Leo et al., 2020; Reddy et al., 2017; Pitkänen et al., 2007; Pitkänen et al., 2016; He et al., 2025; Karhunen et al., 2005). Accordingly, the current systematic review will provide an update to the literature and will be the first to include a meta-analysis of the relationship between PSS and outcomes (e.g., lesion volume). This will help determine the adequacy of preclinical models for replicating the patient experience and identify new avenues for therapeutic research. Moreover, this review will assess the quality of preclinical studies on PSS and make recommendations for improvement and future directions. Ultimately, the goal of this study is to provide up-to-date evidence of PSS incidence and consequences in preclinical literature as well as recommendations for future research.

## 2 Methods

### 2.1 Search strategy

The review and meta-analysis was pre-registered with the International Prospective Register of Systematic Reviews (Prospero, ID: CRD42023443415). We performed a systematic search of the Embase, Medline, Scopus, and Web of Science databases on June 14, 2024, and updated it on May 30, 2025, due to delays in the analysis. The search aimed to identify studies assessing seizures and outcomes in animal models of stroke.

The following search terms were used: (intracerebral h*morrhage OR intracranial h*morrhage OR ICH OR h*morrhagic stroke OR intraparenchymal h*morrhage) OR (focal isch*mial* OR stroke OR isch*mi* stroke OR mcao OR middle cerebral artery occlusion OR thromboembolic stroke OR atherothrombotic stroke OR occlusive stroke) OR (subarachnoid h*morrhage OR SAH OR subarachnoid bleed* OR SAB) AND (seizure* OR epilep* OR epilept*) AND (experimental OR pre-clinical OR rabbit* OR animal model OR animal* OR mouse OR mice OR rodents* OR rat* OR murine* OR hamster* OR pig* OR piglet* OR swine OR horse* OR equine OR cow* OR cattle OR bovine OR goat* OR sheep OR lamb* OR ovine OR monkey* OR primate* OR non-human OR marmoset* OR murine* OR canine* OR dog* OR feline OR cat* OR zebrafish).

### 2.2 Eligibility criteria

The review was limited to original, peer-reviewed articles published in English after 1999. This timeframe was selected to align with the publication of the 1999 STAIR guidelines, increasing the likelihood of adherence to contemporary methodological standards like the CAMARADES checklist (Fisher et al., 2009; Macleod et al., 2004). A secondary, unpublished search for articles pre-dating 1999 yielded no eligible results.

The primary objective of this systematic review was to determine the relationship between seizures following stroke induction and outcomes. Therefore, inclusion criteria focused on studies that measured seizures and reported on outcomes such as lesion volume, neurological deficit scores, behavior, edema, and markers of inflammation in animal models of stroke without intervention (e.g., neuroprotective treatment). Studies were excluded if they were clinical studies, involved neonatal or childhood models, induced seizures (e.g., kainic acid), or focused solely on the impact of a treatment (e.g., anticonvulsant) without providing seizure outcome for the vehicle/control group.

### 2.3 Data extraction

Five reviewers (ACK, AVG, KDLC, MMO, NKNW) independently screened studies using Covidence software (Veritas Health Innovation, Melbourne, Australia) (Muka et al., 2020). Conflicts during screening were resolved by the senior author (ACK). Data extracted related to study details (e.g., authors, publication year), stroke model, seizure characteristics, and descriptive statistics for the outcomes (e.g., lesion volume, NDS, etc.). For studies with multiple time points, data from the latest timepoint were selected. To obtain missing or supplementary data, we contacted authors by email to request non-explicitly reported data on the seizure-outcome relationship, allowing a six-week response period. Endpoint data from graphs and tables were converted and calculated from the published article or supplementary data where necessary.

### 2.4 Risk of bias and study quality assessment

Because our meta-analysis focused on the relationship between PSS and stroke outcomes rather than a specific intervention, we adapted commonly used quality assessment and risk-of-bias checklists and guidelines (e.g., CAMARADES, SYRCLE, STAIR) to fit this non-interventional objective (Fisher et al., 2009; Macleod et al., 2004; Hooijmans et al., 2014). Our assessment checklist included the following criteria: (1) temperature control during stroke, (2) blinding of outcome assessment, (3) random outcome assessment (e.g., randomly selecting animals for outcome assessment), (4) use of aged animals and/or animals with comorbidities, (5) use of animals of both sexes, (6) *a priori* sample size calculations, (7) report of attrition/exclusions, (8) selective outcome reporting (e.g., not reporting on all outcomes measured), (9) statistical bias (e.g., using parametric tests for non-parametric data), and (10) conflict of interests statements.

### 2.5 Statistical analysis

Statistical analyses were performed using Comprehensive Meta-Analysis (CMA) software (Version 4) (Biostat Inc., Englewood, USA). We conducted random-effects meta-analyses for endpoints with sufficient data and assessed publication bias using a funnel plot and Egger’s regression test and the trim-and-fill method. Standardized mean differences are reported as Hedge’s G with 95% confidence intervals (CI). Positive values indicated larger outcomes in the seizure group and negative values indicated larger outcomes in the no-seizure group (Vesterinen et al., 2014). Heterogeneity was calculated using the Q value and represented using the *I*^2^ statistic. Statistical analyses were considered significant when *p* < 0.05. Graphing and visual presentations were prepared using Covidence (PRISMA flowchart), CMA (forest and funnel plots), the robvis tool (Mcguinness and Higgins, 2021) (risk of bias table), and Prism Graphpad software version 9.4.0 (GraphPad Software LLC, San Diego, CA) for other figures.

## 3 Results

### 3.1 Search results

**Figure 1:**
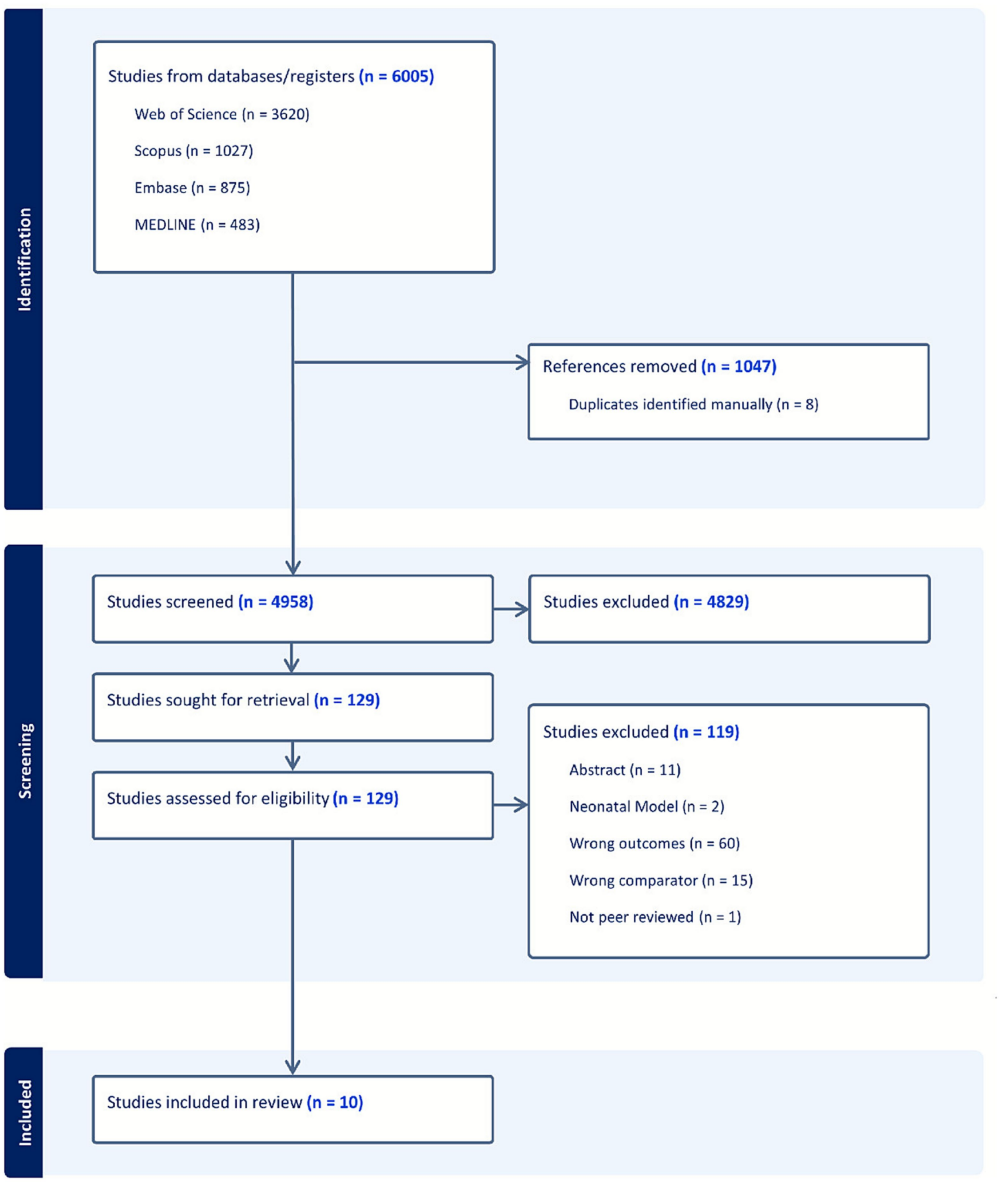
PRISMA flow diagram. This diagram details the study selection process for the systematic review and meta-analysis on PSS in animal models. It illustrates the number of records identified from systematic database searches (Embase, Medline, Scopus, and Web of Science), screened, retrieved for full-text review, and ultimately included in the qualitative synthesis (*n* = 10) and quantitative meta-analysis (*n* = 8 for the lesion volume endpoint).

A total of 6,005 articles were initially retrieved for screening and review (Figure 1). Following a full-text review based on exclusion and inclusion criteria, 17 articles were selected for data extraction. Further correspondence with the authors of seven of these studies revealed that the data necessary to compare outcomes between animals with and without seizures was inaccessible, leading to their exclusion. This process resulted in a final set of 10 articles included in the systematic review (Karhunen et al., 2007; Klahr et al., 2015; Klahr et al., 2016; Germonpré et al., 2020; Germonpré et al., 2021; Jin et al., 2024; Wilkinson et al., 2020; Lu et al., 2013; Hu et al., 2024; Tsai et al., 2011). Of these, only eight were eligible for meta-analysis, which was limited to the single endpoint of lesion volume. While enough studies reported neurological deficit scores (NDS) to permit a pooled analysis, a meta-analysis was not performed due to the non-parametric and ordinal nature of the NDS assessment, which precluded robust quantitative pooling.

To informally evaluate the prevalence of preclinical research of seizures after stroke, a VOSviewer analysis was conducted on the 500 most common co-occurring keywords from a PubMed search of animal models of ischemia and hemorrhagic stroke (Van Eck and Waltman, 2014). The resulting visualization revealed the absence of the terms “epilepsy” and “seizure” (see Supplementary Figure 1), which dramatically highlights the scarcity of studies specifically investigating PSS and PSE in animal models of stroke.

### 3.2 Characteristics of included studies

The 10 included studies were evenly divided, with five (50%) employing models of focal ischemia and five (50%) using ICH models (see Supplementary Table 1 for study details). Rats were the predominant species (80%), consisting of seven studies with Sprague–Dawley rats and one with Wistar albino rats, while the remaining two studies (20%) used mice (C57BL/6 J and C57BL/6 N).

**Figure 2:**
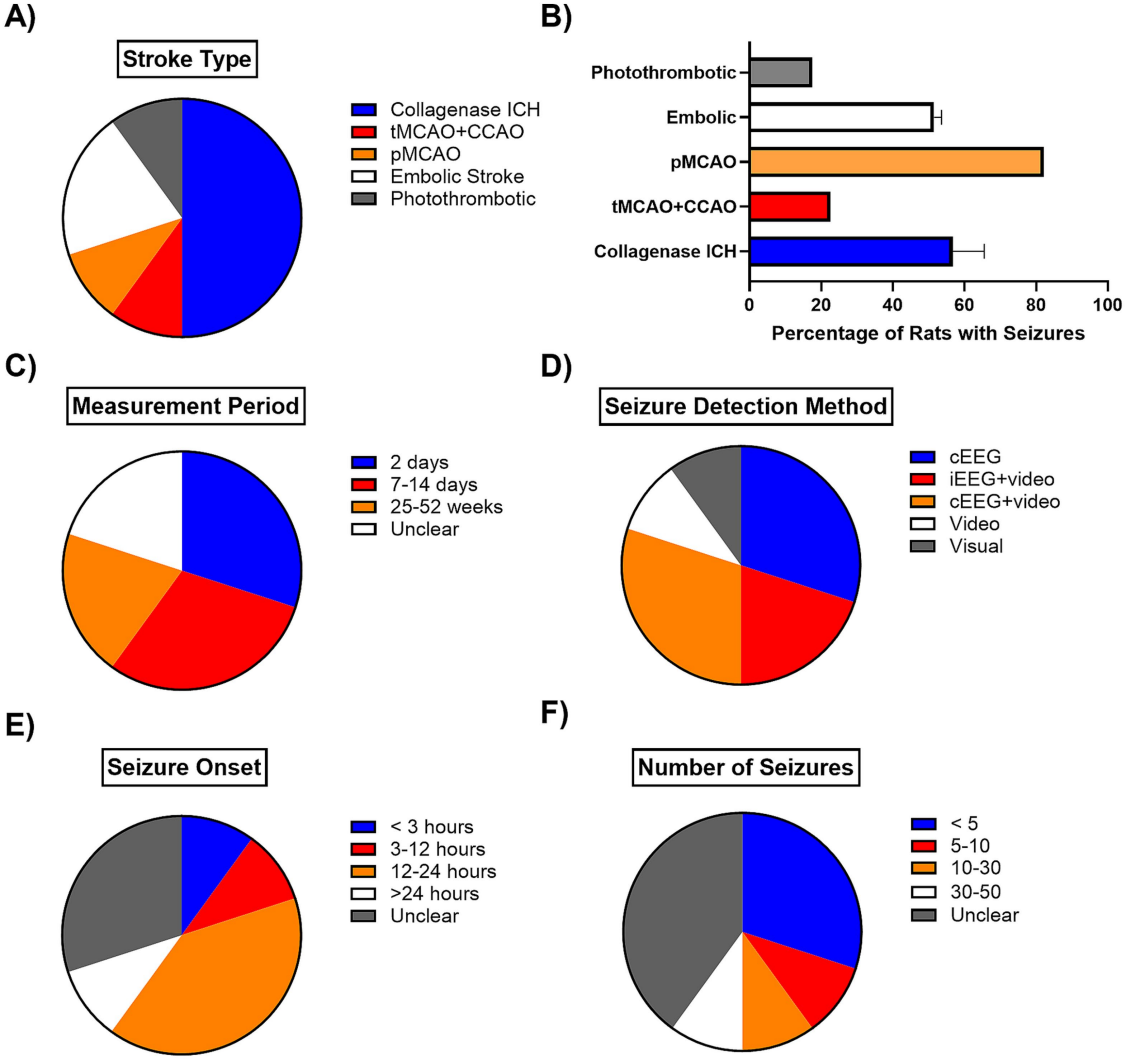
Characteristics of included studies. A summary of the key methodological and outcome details across the 10 included animal studies. This figure details parameters such as (**A**) the number of studies using specific animal models of stroke, (**B**) the percentage of rats with seizures for each specific model, (**C**) the number of studies measuring PSS during the acute (within 2 days), sub-acute (7-14 days) and chronic phase (25+ weeks), (**D**) the number of studies using specific seizure detection methods, (**E**) the number of studies reporting different timings for PSS seizure onset and (**F**) the average number of seizures rodents had.

All ICH studies employed the collagenase model (Figure 2A, Supplementary Table 1). In contrast, the IS studies demonstrated a greater diversity of models, including transient middle cerebral artery occlusion (tMCAO, 60 min) with common carotid artery occlusion (CCAO, 10%), permanent middle cerebral artery occlusion (10%), embolic stroke models (20%), and photothrombotic stroke (10%). The reported percentage of rats experiencing seizures ranged from 17.50 to 82.14% in the focal ischemia models and from 45.45 to 66.67% in the ICH models (Figure 2B, Supplementary Table 1).

The timeframe for seizure measurement varied widely, from 2 days to as long as 52 weeks (Figure 2C, Supplementary Table 1). For seizure detection, only three studies (30%) used continuous EEG (cEEG) with video monitoring, while another three (30%) used cEEG without video (Figure 2D). Two studies (20%) used intermittent EEG (iEEG) with video, and the remaining 20% relied solely on visual observation. Notably, 40% of the studies did not use cEEG monitoring at all.

Reported seizure outcomes demonstrated significant variability. The most frequently measured were the number of animals with seizures (90%), seizure duration (70%), seizure latency or onset (70%), and the total number of seizures (60%) (Figures 2E,F, Supplementary Table 1). On average, seizures commonly started within 24 h from stroke onset (60%), though Germonpré et al. (2021) reported seizure onsets after 90 days post-ICH in two animals (Figure 2E).

The total number of seizures varied greatly, with most rats experiencing, on average, less than 10 seizures (40%). However, 40% of the studies did not report this endpoint. Overall, assessments of seizure severity were less consistent, including laterality (40%), the Racine score (30%) for studies with video monitoring, and other, less common outcomes like power, coherence increases, and periodic epileptiform discharges. Altogether, poor data availability, accompanied by small sample sizes, and lack of reporting of the number of animals prevented any meta-regression analyses.

### 3.3 Outcome measures

**Figure 3:**
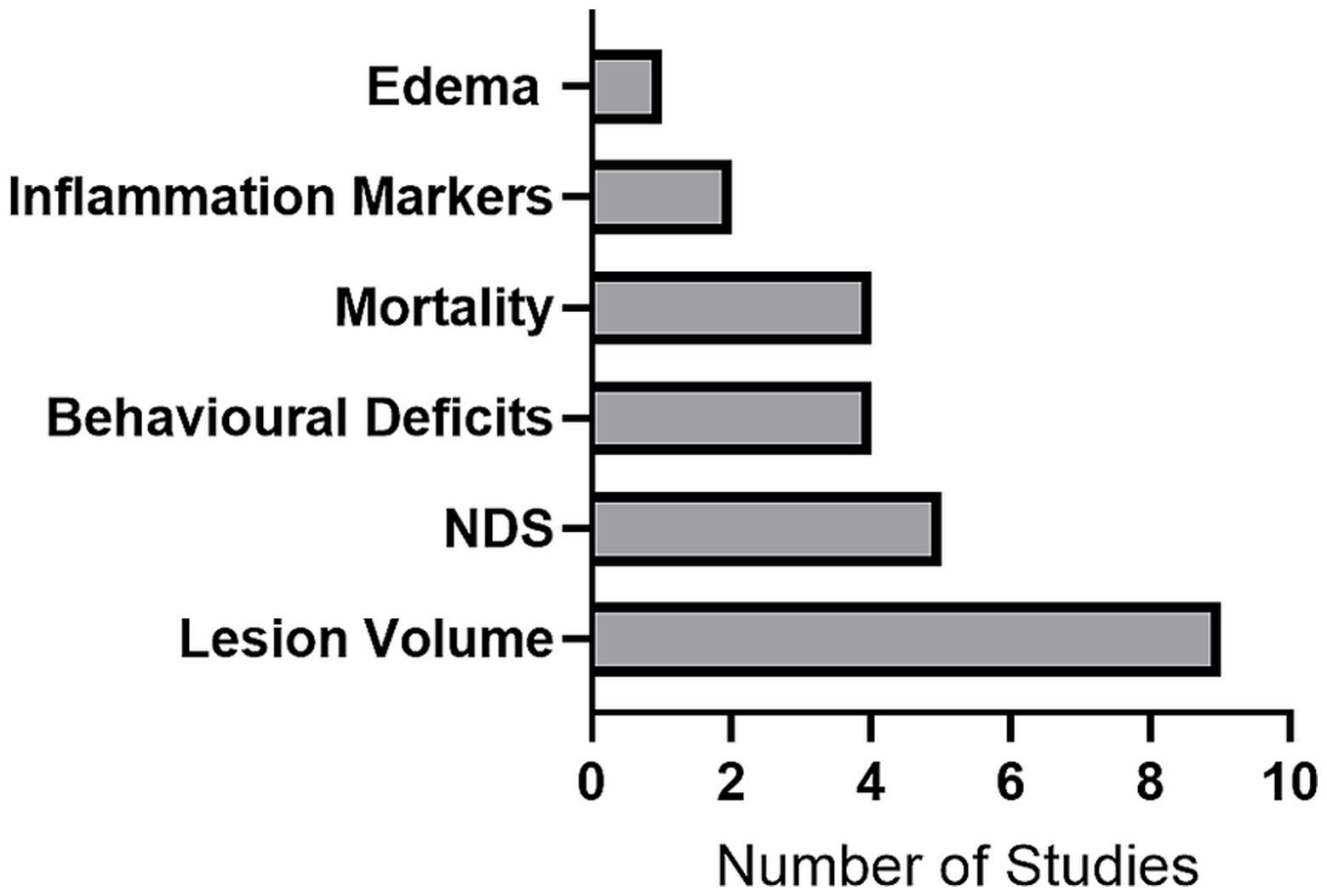
Distribution of outcome measures. The frequency of different endpoints assessed across the included animal studies indicates that lesion volume was the most commonly assessed endpoint while edema was the least measured.

Lesion volume was the most assessed endpoint, measured in 90% of studies, and the only outcome for which a meta-analysis was feasible (Figure 3). However, the time of euthanasia (i.e., the point at which lesion volume was measured) varied widely, ranging from 48 h to 52 weeks. This information was not provided in two studies (20%) (Supplementary Table 1). Other frequently assessed endpoints included neurological deficit scores (NDS, 50%), behavioral assessments (40%), and mortality (40%, Figure 2F). Two studies (20%) reported on the expression of inflammatory markers, while only one study (10%) assessed edema.

Lesion volume location was measured in three studies, revealing patterns associated with seizure incidence. Germonpré et al. (2020) found that larger anterior–posterior spanning of an ICH was associated with the presence of seizures. Importantly, in a follow-up study, they reported that the involvement of the piriform cortex led to higher incidence of acute seizures (Germonpre et al., 2021). Similarly, Jin et al. (2024) reported that convulsive seizures as well as mortality were present mainly when the hippocampus was affected in a more severe MCAO+CCAO model of IS.

Interestingly, the relationship between lesion size and PSS differed markedly by stroke type (Table 1). Individual ICH studies consistently reported no relationship between lesion volume and seizures. In contrast, among the four studies using focal ischemia models that measured lesion volume, three observed that seizures were associated with larger lesion sizes.

**Table 1:** Summary of the relationship between seizures and outcomes in animal models of stroke.

Author & Year (Stroke type)	Lesion volume	NDS	Behavioral deficits	Inflammation markers	Edema
Germonpré et al. (2020) (ICH)	∅	∅	−	N/A	N/A
Germonpré et al. (2021) (ICH)	∅	N/A	∅	∅	N/A
Hu et al. (2024) (Focal Ischemia)	+	+	+	+	N/A
Jin et al. (2024) (Focal Ischemia)	∅	+	N/A	N/A	∅
Karhunen et al. (2007) (Focal Ischemia)	N/A	N/A	+	N/A	N/A
Klahr et al. (2015) (ICH)	∅	N/A	N/A	N/A	N/A
Klahr et al. (2016) (ICH)	∅	∅	N/A	N/A	N/A
Lu et al. (2013) (Focal Ischemia)	+	N/A	N/A	N/A	N/A
Tsai et al. (2011) (Focal Ischemia)	+	+	N/A	N/A	N/A
Wilkinson et al. (2020) (ICH)	∅	N/A	N/A	N/A	N/A

The null sign depicts (∅) no changes, a negative sign (−) a decrease, and a positive sign (+) an increase in the outcome (e.g., lesion volume) when seizures were present. Not applicable (N/A) depicts that the variable was not measured.

A similar pattern was mirrored in the NDS results: no ICH studies found that seizures were linked to worsened NDS, but three of the focal ischemia studies that assessed NDS reported that PSS were associated with poorer neurological outcomes. Behavioral deficits were assessed in only two ICH studies, yielding mostly null results, except for one finding that rats with seizures had less bias in the use of the non-paretic limb in a cylinder task (Supplementary Table 1). The two focal ischemia studies assessing behavior revealed that seizures worsened performance in rotarod, beam walking, and spatial memory tasks.

Finally, one ICH study assessed the inflammatory markers Iba1, GFAP, and vimentin and reported no relationship with seizures. However, one focal ischemia study reported an association between inflammation (Hsp90aa1 and JUN markers) and seizures. Lastly, only one focal ischemia study measured edema by hemispheric swelling, reporting no relationship to seizures.

### 3.4 Meta-analysis of lesion volume

Of the nine studies assessing lesion volume, one was excluded due to having only two animals in the non-seizure group, leaving eight studies eligible for analysis. The overall pooled effect indicated that lesion volume was not larger in the seizure group, despite a statistical trend (Hedge’s G = 0.610, [0.000, 1.219], *p* = 0.050). This finding was complicated by substantial and significant heterogeneity (*I*^2^ = 55.994, *Q* = 15.907, df = 7, *p* = 0.026), which suggests that the overall pooled effect does not reliably represent the association between seizure incidence and lesion volume. Given that individual studies carried out in animal models of focal ischemia suggest an association between seizures and lesion volume, we carried out a subgroup analysis.

**Figure 4:**
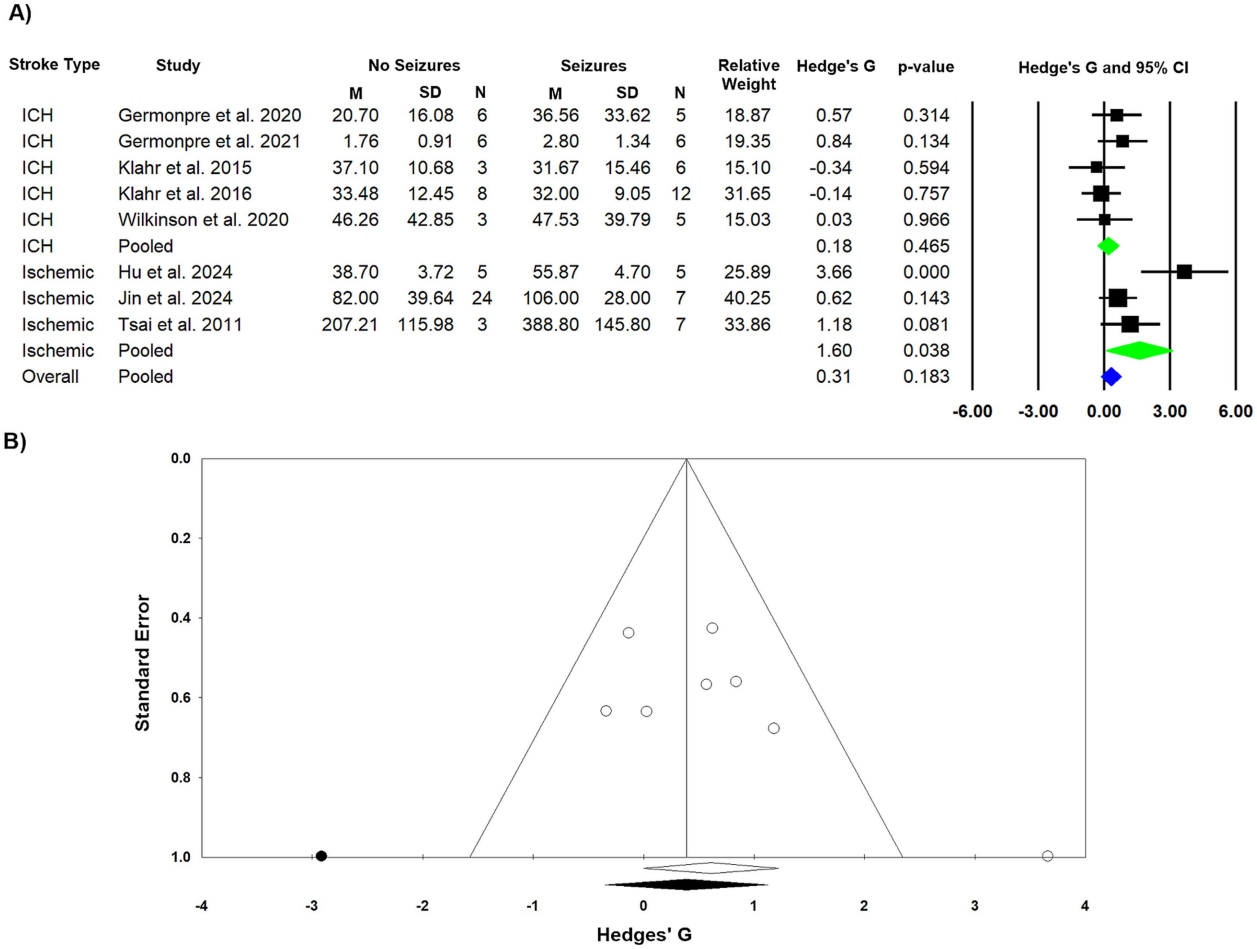
Meta-analysis of lesion volume and publication bias. **(A)** Forest plot showing the pooled standardized mean difference (Hedge’s G) for the effect of PSS on lesion volume, presented with 95% confidence intervals (CI). The subgroup analysis by stroke type indicates that PSS are associated with a significantly larger lesion volume in focal ischemia models but show no significant association in intracerebral hemorrhage (ICH) models. **(B)** Funnel plot assessing potential publication bias for the lesion volume meta-analysis. The plot suggests significant asymmetry, which is indicative of potential publication bias or small-study effects.

A subgroup analysis by stroke type (Figure 4A), indicated that seizures were not associated with larger lesion volumes post ICH (Hedge’s G = 0.180, 95% CI [−0.303, 0.663], *p* = 0.468). There was no significant heterogeneity among the ICH studies (*I*^2^ = 0.0%, *Q* = 3.102, df = 4, *p* = 0.541). As predicted from the individual studies, the pooled effect in the focal ischemia subgroup indicated a significant increased lesion size in the seizure group (Hedge’s G = 1.598, 95% CI [0.091, 3.104], *p* = 0.038), although there was significant heterogeneity within this subgroup (*I*^2^ = 74.506, *Q* = 7.845, df = 2, *p* = 0.020), which indicates that this association should be considered with caution. Despite the significant finding in the focal ischemia subgroup, the overall effect of the subgroup analysis was not significant (Hedge’s G = 0.312, 95% CI [−0.148, 0.771], *p* = 0.183).

Lastly, we assessed potential publication bias using a funnel plot (Figure 4B). The Egger’s linear regression test indicated significant asymmetry (Intercept = 3.863, 95% CI [−0.886, 8.611], *p* = 0.0468, *p* = 0.015). To adjust for this, the trim-and-fill method imputed a single missing study on the left side of the funnel plot. While this asymmetry is suggestive of publication bias, it could also be influenced by other factors, such as true differences in effect sizes between studies of varying sizes. Still, it raises concerns about the potential for small-study effects in our meta-analysis.

### 3.5 Risk of bias and study quality assessment

**Figure 5:**
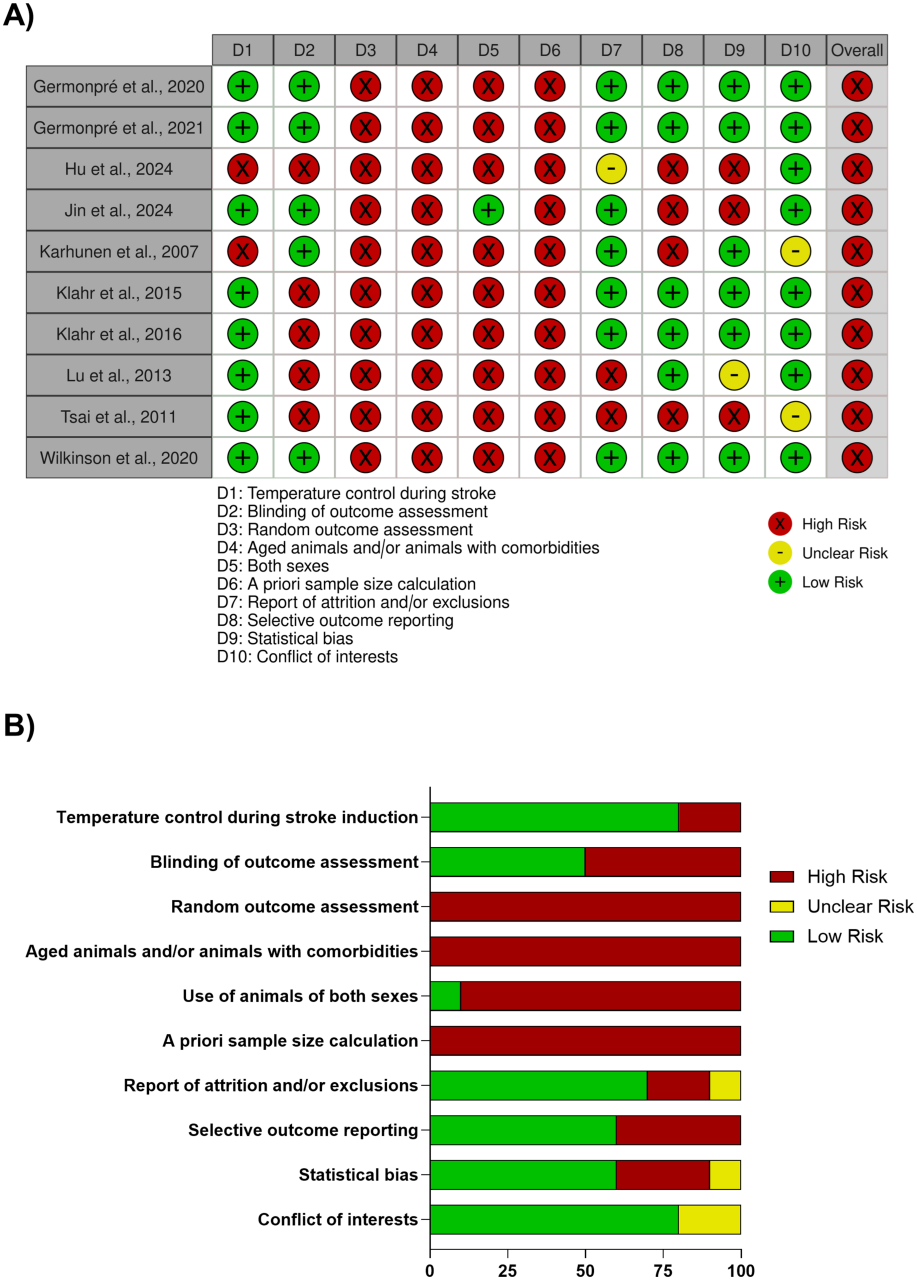
Summary of risk of bias assessment. **(A)** Risk of bias summary showing the overall assessment for each of the included studies. Every study was categorized as having an overall high risk of bias due to limitations in at least four domains. **(B)** Risk of bias graph illustrating the percentage of studies that met or failed to meet each of the 10 specific quality criteria, adapted for this non-interventional review. Notably, no study met critical criteria for methodological rigor: random outcome assessment, use of aged animals and/or animals with comorbidities, or *a priori* sample size calculations.

An evaluation of all included studies revealed that each had at least four dimensions rated as high risk, resulting in an overall assessment of high risk of bias for every study (Figure 5A). Consequently, the meta-analysis should be interpreted with caution. In summary, no study met three critical criteria for methodological rigor: random outcome assessment, use of aged animals and/or animals with comorbidities, and *a priori* sample size calculations (Figure 5B). The absence of these factors significantly impacts the generalizability and translatability of the findings. This highlights a critical need for improved methodological rigor in future translational studies. Another notable translational limitation across the included studies was the predominant use of male animals, as only one study (10%) included both sexes.

While some studies demonstrated good methodological practices, including maintaining normothermia during stroke induction (80%), reporting on attritions and exclusions (70%), and disclosing conflicts of interest (80%), inconsistencies remain. Specifically, only half of the studies (50%) assessed outcomes blindly, and a significant number engaged in selective outcome reporting (60%) or used statistical analyses inappropriately (60%).

## 4 Discussion

The findings of our systematic review and meta-analysis underscore the significant translational and methodological limitations within the existing preclinical literature on PSS. Our comprehensive search, which initially yielded over 6,000 articles, was ultimately narrowed down to 10 studies for qualitative synthesis and eight for quantitative meta-analysis. The limited inclusion of studies was a result of inaccessible raw data, compounded by issues with clear reporting. Also, PSS is heavily underinvestigated in preclinical models, as evidenced by the VOSviewer analysis. The included studies, though informative, were highly heterogeneous in methodology, with considerable variability in stroke models, species, seizure detection methods, and outcome measures. The meta-analysis results suggest that PSS are associated with larger lesion volumes in focal ischemia models, but not in ICH. These findings must be interpreted with caution due to the substantial risk of bias across all included studies. Ultimately, the outcomes of this review underscore a critical need for improved methodological rigor to enhance the translatability of preclinical research on PSS.

### 4.1 Preclinical findings and translational gaps in epidemiology

The pooled analysis for lesion volume did not yield a statistically significant overall association with PSS. However, this result is rendered inconclusive by the substantial and significant heterogeneity observed across studies, likely reflecting differences between ischemic and hemorrhagic models. Subgroup analysis by stroke type provides a more clinically meaningful interpretation of the association between PSS and lesion volume. While pooled data from ICH models showed no significant association between seizures and larger lesion volumes, consistent with individual studies, the focal ischemia subgroup demonstrated a link between seizure incidence and larger lesion sizes. Still, the focal ischemia subgroup analysis indicated significant heterogeneity, potentially due to the use of different models and diverse measurement timings. Unfortunately, due to the small sample size, these variables were not included in the analysis. The lack of association between hematoma volume and PSS in ICH models is an interesting preclinical observation warranting further study, particularly as clinical evidence links ICH volume to higher seizure incidence (Haapaniemi et al., 2014; Biffi et al., 2016). Outside of the meta-analysis, individual focal ischemia studies indicated an association between PSS and more severe NDS, although it remains unclear whether neurological deficits were driven by lesion volume itself or by the occurrence of PSS. Larger infarct size is a known predictor of both early (≤7 days) and late (>7 days) PSS in patients, and is strongly associated with worse functional and behavioral outcomes, including higher mortality and disability (Galovic et al., 2018).

Our review, and others, have also identified a significant gap in the PSS literature regarding lesion location and seizure incidence in animal models (Pitkänen et al., 2016). Unlike in patients, where cortical location is a well-established risk factor for both PSS and PSE, the included studies provided minimal data on this variable. In patients, IS affecting the cerebral cortex or temporal lobe confer a significantly higher risk of seizure development due to the epileptogenicity of these regions. Three studies included in our review noted that temporal lobe and hippocampal involvement were related to higher risk of seizure incidence (Germonpré et al., 2020; Germonpré et al., 2021; Jin et al., 2024). These findings are consistent with the well-established clinical observation that cortical and temporal lobe involvement strongly increases seizure incidence (Haapaniemi et al., 2014; Galovic et al., 2018). However, most animal studies in our meta-analysis did not report lesion location, precluding direct comparison. Given the importance of lesion site in clinical seizure prediction, its omission in preclinical models represents a major translational gap that warrants further investigation.

While both IS and ICH can precipitate seizures, in patients ICH presents the most significant risk of PSS and PSE due to the epileptogenic effects of blood components like iron and thrombin (Serafini et al., 2015). Nevertheless, because IS accounts for 65% of all cases, compared with 29% for ICH, a greater absolute number of patients experience PSS and PSE following ischemic brain insults (Feigin et al., 2025; Leo et al., 2020). This disparity highlights the importance of clarifying the anatomical origin of seizure generation in epileptogenesis, a critical dimension missing from most included studies except the ones using ICH models (Klahr et al., 2015; Klahr et al., 2016; Germonpré et al., 2020; Wilkinson et al., 2020). Clinical evidence overwhelmingly suggests that PSS are generated in the ipsilesional hemisphere, with activity potentially propagating contralaterally, and that cortical involvement is a primary risk factor for chronic PSE (Dziadkowiak et al., 2021; De Reuck et al., 2007; Zhang et al., 2014). However, this rule may be circumvented by the strong chemical irritation inherent to ICH, as one study found a subcortical hematoma location to be an independent risk factor for PSE development, underscoring that the underlying pathology can sometimes override typical anatomical epileptogenicity (Lahti et al., 2017). In contrast, our findings in animal models showed a comparable or greater seizure incidence in focal ischemia models than in ICH models, diverging from the clinical literature. This discrepancy may reflect differences in the stroke induction methods, species, and lesion sites (He et al., 2025). Another factor may be the severe nature of the IS models used.

Beyond differences in stroke pathology and lesion characteristics, another critical translational gap lies in the detection and characterization of seizures. In animal studies, PSS are almost exclusively detected with cEEG or video-EEG telemetry, allowing for precise capture of both convulsive and nonconvulsive events. In contrast, clinical seizure detection remains largely reliant on clinical observation or intermittent EEG, which underestimates true incidence. Recent human studies employing cEEG have demonstrated that a substantial proportion of ischemic and hemorrhagic stroke patients experience subclinical or electrographic seizures, often without overt clinical correlates (Biffi et al., 2016; Rossetti et al., 2020; Claassen et al., 2004; Peter-Derex et al., 2022). For instance, subclinical events are detected in approximately 10–15% of unselected ischemic-stroke patients and up to 40% of those with neurological deterioration (Scoppettuolo et al., 2019). Moreover, the Prevention of Epileptic Seizures in the Acute Phase of Intracerebral Hemorrhage (PEACH) trial, which assessed prophylactic administration of levetiracetam, reported that 43% of ICH patients in the placebo group had seizures when monitored with cEEG, a rate comparable to that seen in the ICH studies included in this review (Peter-Derex et al., 2022).

Translational understanding requires contextualizing seizure characteristics across defined clinical stroke phases. Most of the included studies (60%) reported seizure onset within 24 h from stroke induction, indicating that these models primarily capture the highly epileptogenic phenomena associated with the hyperacute and acute clinical periods. Preclinical studies detailing this hyperacute seizure generation have identified acute seizures via cEEG monitoring (Pitkänen et al., 2007; He et al., 2025; Karhunen et al., 2005; García-Peña et al., 2023), however there is limited insight into long-term PSE prevalence and its protracted consequences. Others have noted that early seizures in focal ischemic models rarely predict chronic epilepsy, although this has been confounded by the lack of prolonged monitoring, underscoring the need for extended observation (Karhunen et al., 2005). In patients, acute seizures carry a 33% recurrence risk, whereas unprovoked seizures carry a 71% risk (Nandan et al., 2023). This discrepancy in monitoring helps explain the higher seizure rates observed in preclinical models compared with patient cohorts and underscores the importance of standardizing continuous monitoring across species. To contextualize these methodological and temporal differences, Table 2 compares the incidence, timing, and lesion-related predictors of seizures in animal models versus clinical stroke populations, highlighting distinctions between clinical and subclinical detection and between early and late seizure occurrence.

**Table 2:** Comparative features of PSS in experimental stroke models and human stroke population.

Feature	Collagenase ICH (animal)	Focal ischemia (animal)	ICH patients	Ischemic stroke patients
Seizure incidence	45–67%; frequent early seizures, cEEG telemetry + video up to 180 days showed 50% seizure incidence	17.5–82%, model-dependent (e.g., lowest PSS incidence using photothrombotic, highest pMCAO 82%)	≈11% early clinical PSS, up to 40% subclinical PSS; ≈9% late (>7d, 2y); 7.1% (1y) → 11.8% (5y) (Biffi et al., 2016; Peter-Derex et al., 2022)	≈3.3% early clinical PSS; 10–15% subclinical (Rossetti et al., 2020; Claassen et al., 2004); ≈4% (1y) → 8% (5y) late
Timing (definitions & patterns)	Mostly within 24 h (hyperacute); delayed events reported up to 180 days	Mostly within 24 h (hyperacute)	Early PSS more common than late; cumulative late risk increases over years	Late PSS more common long-term
Lesion/bleed volume association	No clear relationship between hematoma volume and seizures	Larger infarcts predict PSS (Lu et al., 2013; Tsai et al., 2011)	Larger hematomas increase seizure risk (Haapaniemi et al., 2014; Biffi et al., 2016)	Larger cortical infarcts increase early and late seizure risk (Galovic et al., 2018)
Lesion location	Cortical/piriform involvement increases risk (Germonpré et al., 2021)	Cortical, hippocampal, and temporal involvement increase risk (Jin et al., 2024; Lu et al., 2013)	Cortical and lobar ICHs most epileptogenic (Haapaniemi et al., 2014; Biffi et al., 2016)	Cortical and temporal infarcts most strongly linked to seizures (Galovic et al., 2018; Serafini et al., 2015; Dziadkowiak et al., 2021)
Outcome associations	Mixed; mostly NDS not associated with PSS	Seizures associated with larger infarcts and worse outcomes (Lu et al., 2013; Tsai et al., 2011)	Seizures linked to higher disability and mortality (Haapaniemi et al., 2014; Biffi et al., 2016)	Seizures predict poorer functional recovery and mortality (Galovic et al., 2018; Serafini et al., 2015)

Clinically, “early seizures” are defined as those occurring within 7 days of stroke onset, and “late seizures” as those occurring thereafter. In animal models, collagenase ICH studies report high PSS incidence with both acute and delayed events, including cEEG telemetry up to 180 days (Klahr et al., 2015; Klahr et al., 2016; Germonpré et al., 2020; Germonpré et al., 2021; Wilkinson et al., 2020). Focal ischemia show variable PSS rates depending on injury severity and cortical involvement (Karhunen et al., 2007; Jin et al., 2024; Lu et al., 2013; Hu et al., 2024; Tsai et al., 2011). In patients, early seizures occur more frequently after hemorrhagic stroke (Haapaniemi et al., 2014; Biffi et al., 2016; Lahti et al., 2017; Peter-Derex et al., 2022) than ischemic stroke (Galovic et al., 2018; Zhang et al., 2014). Larger and cortical lesions increase seizure risk in both ischemic and hemorrhagic stroke patients (Haapaniemi et al., 2014; Biffi et al., 2016; Galovic et al., 2018; Zhang et al., 2014; Lahti et al., 2017).

### 4.2 Pathophysiological mechanisms of post-stroke epileptogenesis

The process of post-stroke epileptogenesis is driven by distinct acute and chronic pathological cascades following ischemic or hemorrhagic stroke. According to the International League Against Epilepsy (ILAE), acute PSS, often linked to transient factors with a low recurrence risk, occur within the first seven days post-stroke (Reddy et al., 2017; Serafini et al., 2015; Doria and Forgacs, 2019; Fisher, 2017; Yang et al., 2018). These early PSS are primarily triggered by perilesional hyperexcitability stemming from excitotoxicity, where the massive release of glutamate from damaged cells hyperstimulates N-methyl-D-aspartate receptors (NMDARs), leading to profound ionic dysregulation and neuronal depolarization (Yang et al., 2018). Another pathophysiological mechanism increasingly recognized to influence post-stroke excitability is cortical spreading depolarization (CSD), a propagating wave of near-complete neuronal and glial depolarization that travels across the peri-infarct cortex. While recurrent CSDs can aggravate ischemic injury through metabolic stress, they have also been shown to transiently suppress epileptiform discharges, acting as an intrinsic antiseizure mechanism during the acute post-stroke phase (Dreier, 2011; Von Bornstädt et al., 2015). Understanding how CSD interacts with evolving excitatory–inhibitory balance could help explain variability in seizure susceptibility between stroke types and guide future mechanistic investigations.

This acute phase is significantly exacerbated by generalized inflammatory cascades and blood–brain barrier disruption, which allows pro-epileptogenic cytokines and serum components to lower the neuronal seizure threshold. Crucially, the acute rise in excitability following ICH is intensified by the presence of thrombin, which directly enhances NMDAR function and acute seizure susceptibility. Over time, unprovoked seizures arise from enduring pathophysiological changes (Nandan et al., 2023; Leo et al., 2020; Galovic et al., 2018; Serafini et al., 2015; Zhao et al., 2018). Specifically, the chronic phase of epileptogenesis is characterized by maladaptive plasticity and gliosis, where reactive astrocytes form a scar that structurally and functionally rewires the neuronal network, often accompanied by a net loss of GABAergic inhibitory tone in the peri-lesional zone (Yang et al., 2018). However, the mechanism leading to chronic PSE diverges significantly in hemorrhage: ICH has a uniquely high epileptogenic potential attributed to the chronic neurotoxicity of hemosiderin deposition (Yang et al., 2018). The ionic iron released during hemoglobin breakdown catalyzes the production of damaging hydroxyl radicals, leading to chronic chemical irritation and cortical superficial siderosis, which is strongly implicated as an irreversible step in establishing a permanent epileptic focus (Yang et al., 2018).

### 4.3 Post-stroke seizures, functional outcome, and treatment considerations

Once the pathophysiology of PSS in animal models is further understood, the next main focus should be assessing the long-term effects of PSS on outcome and post-stroke recovery (Karhunen et al., 2005). There is growing evidence of a bidirectional relationship between seizures and neurodegeneration, where seizures can worsen metabolic stress and injury, and neurodegeneration can predispose to seizures. Clinical studies like Kumral et al. (2013) have reported that patients with PSS often experience long-term neurological worsening and diffusion changes consistent with injury, and that even a single PSS can sometimes cause persistent decline (Bogousslavsky et al., 1992; De Reuck et al., 2006; Bryndziar et al., 2016). A recent meta-analysis further confirmed that PSS and PSE are strongly associated with higher mortality and severe disability (Misra et al., 2023). Collectively, this evidence highlights the urgent need for seizure prevention (Misra et al., 2023).

Clinical stroke guidelines continue to recommend against routine prophylactic AED use for primary prevention, citing limited benefit and potential harm (Heran et al., 2022; Holtkamp et al., 2017; Wolcott et al., 2025). However, these questions could be more readily addressed in preclinical models, which allow for controlled, long-term studies with cEEG monitoring not as feasible in patients. Williams et al. (2004) provided rare preclinical data suggesting that fosphenytoin, valproate, ethosuximide, and gabapentin may reduce seizure incidence, infarct size, and mortality after focal ischemia. Although excluded from our analysis due to AED treatment being an exclusion criterion, the study highlights the gap between promising preclinical findings and lack of demonstrated clinical benefit. In practice, AEDs are prescribed only after a seizure, for secondary prophylaxis, with decisions individualized by risk (Heran et al., 2022; Holtkamp et al., 2017; Wolcott et al., 2025). Specifically addressing the clinical relevance of treatment of PSS, the question remains whether this treatment modifies disease progression or improves functional outcomes. While AEDs are effective in controlling seizures, which prevents secondary brain injury and improves a patient’s quality of life, current clinical evidence does not robustly demonstrate that initiating AEDs after a seizure (secondary prophylaxis) independently leads to better long-term neurological recovery or modifies the underlying post-stroke epileptic process (Wolcott et al., 2025). The primary benefit remains symptomatic control, preventing the acute risks of recurrent seizures and status epilepticus, rather than acting as a disease-modifying therapy for stroke-related disability.

### 4.4 Limitations of the current study

Several limitations restrict the interpretation of our findings. The scarcity of eligible studies confined our meta-analysis to a single endpoint—lesion volume. A major factor contributing to the limited scope of preclinical PSS research include the high cost of cEEG hardware and software, which often results in small sample sizes and low statistical power. Our analysis was also limited to English-language, peer-reviewed publications, potentially excluding relevant work. We also excluded neonatal animal models due to their pathophysiology and clinical differences in PSS presentation between infants and adults. In neonates, the brain’s immaturity results in unique neurophysiological properties (e.g., the GABA-A receptor being excitatory), which is unlike the mature circuitry affected in adult post-stroke seizures (PSS) (Chapman et al., 2012; Kang and Kadam, 2014). Furthermore, the primary causes of neonatal seizures are often severe underlying insults like hypoxic–ischemic encephalopathy, which is distinct from the typical adult ischemic or hemorrhagic stroke etiologies (Mondal et al., 2025). Although highly clinically relevant and also unresearched, excluding neonatal models ensures the systematic review maintains strong translational relevance to the mechanisms and therapeutic strategies specifically aimed at the adult stroke patient population, which typically involves PSS arising from a mature brain.

A high risk of bias was evident across all studies, with each failing to meet at least four quality criteria essential for generalizability. No study used random outcome assessment, aged animals and/or animals with comorbidities and none reported the use of *a priori* sample size calculations. The lack of random outcome assessment introduces potential performance and detection bias, meaning the researchers measuring the outcome were not blinded to the seizure status of the animal, which may lead to an overestimation of the effect size (Hooijmans et al., 2014). The predominant use of young, male animals fails to account for the higher lifetime stroke risk in women and the high prevalence of comorbidities in patients (Bushnell et al., 2014). By excluding female animals and models that incorporate age or comorbidities (such as hypertension or diabetes), the preclinical field reports results from an animal population that is poorly representative of the human stroke patient population (Fisher et al., 2009; Lapchak et al., 2013). Others have identified sex effects regarding stroke models and outcomes (Zhang et al., 2019), yet sex differences in PSS incidence is not well understood. Also, the elderly represent the majority of the stroke population, and there have been associations between age and seizure incidence in the clinical literature (Haapaniemi et al., 2014; Liu et al., 2016). This has also been evident in one preclinical study carried out by Kelly et al. (2018) and others in which aged Fischer 344 rats had more PSS, even when they had comparable lesion sizes to the young control group. Therefore, the effect sizes reported here are limited in their generalizability and clinical translatability to the broader population of stroke survivors. Moreover, even though there are differences in the incidence of spontaneous seizure activity in rodent strains (Gu and Dalton, 2017), in this study most rats used were Sprague–Dawley, and there were also not enough studies using diverse mice strain for us to carry out further analyses by strain. For instance, Fischer 344 show spontaneous seizure activity (Kelly et al., 2018), while Sprague Dawley rats do not (Klahr et al., 2015). Unfortunately, despite our efforts, rodent studies were the only ones meeting the inclusion criteria in this study. It is important to emphasize how the use of other species, such as non-human primates and swine, with more similar vasculature and brain anatomy to humans would provide valuable evidence toward our understanding of PSS in patients (He et al., 2025; Nielsen et al., 2024; Faught et al., 1988; Lin et al., 2022; Li et al., 2018). Other species have been used to study epilepsy (Zhu et al., 2023; Bassett et al., 2014; Sanabria et al., 2024), but they are not prevalent in the study of PSS. Furthermore, visual evidence of funnel plot asymmetry and a significant Egger’s regression test further suggest possible publication bias. These issues, together with the lack of standardized reporting, limit the strength of current conclusions (He et al., 2025).

### 4.5 Future directions and conclusion

Based on these findings, we propose several recommendations to improve the rigor and translatability of preclinical research on PSS and PSE. First, researchers should adhere to reporting guidelines such as ARRIVE and consistently apply blinding, randomization, and a priori sample size calculations to ensure adequate power (Fisher et al., 2009; Lapchak et al., 2013; Kilkenny et al., 2011). Second, studies should diversify animal models by including different species (He et al., 2025), both sexes (Jin et al., 2024), aged animals (Wu et al., 2015), and models with common comorbidities, including diabetes (e.g., streptozotocin treated) (Reeson et al., 2016), hypertension (e.g., spontaneously hypertensive rat) (Thakkar et al., 2020; Liao et al., 2013; Wu et al., 2011; González-Darder and Durán-Cabral, 1990), and increased seizure susceptibility by using strains with spontaneous seizures (Fisher et al., 2009; Lapchak et al., 2013; Zhang et al., 2019; Gu and Dalton, 2017; Candelario-Jalil and Paul, 2021). Indeed, factors such as age, hypertension, and diabetes have all been linked to increased seizure incidence in the epilepsy literature (Liu et al., 2016; Ng et al., 1993; Nadeem et al., 2023). Third, seizure monitoring should be standardized, with cEEG monitoring prioritized as the gold standard, despite cost and logistical challenges. Fourth, to fully understand PSE, studies must extend observation beyond the acute phase to capture unprovoked, chronic seizures. Clinically, risk factors for early seizures include stroke severity and location. Although our meta-analysis did not show this in ICH models, future work examining different hematoma sizes and lesion sites may better mirror clinical findings. Lastly, research must clarify whether PSS and PSE contribute to neurodegeneration and whether prophylactic AEDs can improve outcomes. In the TBI literature, seizures are also recognized to worsen long-term deficits. Interestingly, prophylactic AEDs have been used to prevent secondary injury and improve recovery post-TBI (Engel, 2019; Pitkänen et al., 2009). Despite shared mechanisms between TBI and stroke, the stroke field has not yet achieved comparable translational progress (Pitkänen et al., 2007; Galovic et al., 2018; Kumral et al., 2013; Pitkänen et al., 2009). Major stroke guidelines currently recommend against routine prophylactic AED use in patients without seizures, citing insufficient benefit and potential harms (Heran et al., 2022; Holtkamp et al., 2017; Wolcott et al., 2025; Powers et al., 2019).

**Figure 6:**
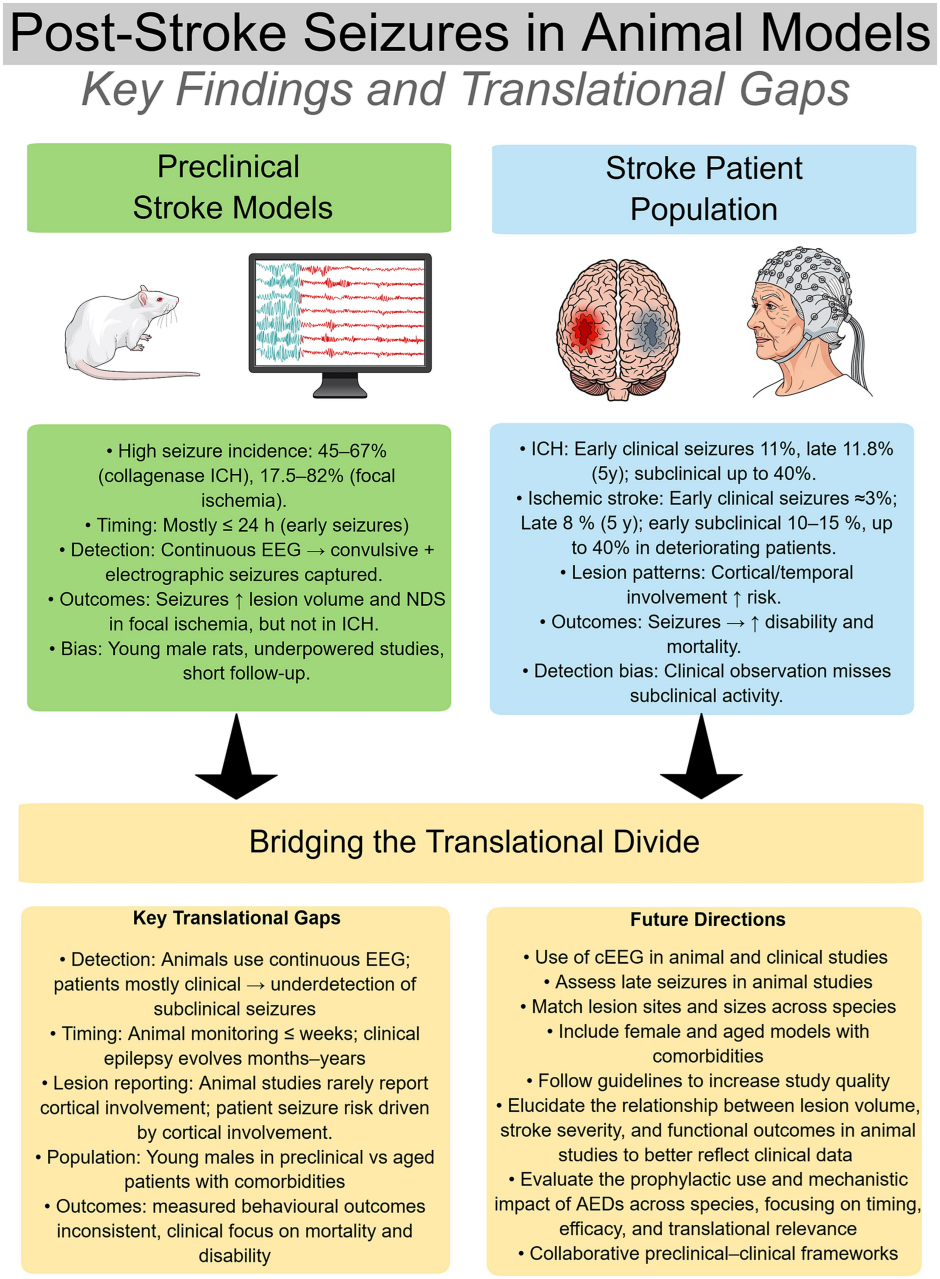
Summary of translational findings and research gaps in post-stroke seizure models and clinical populations. This schematic summarizes major comparative findings between the preclinical studies included in the systematic review and clinical studies, highlighting key methodological differences that limit translational progress. Animal models (left) commonly report high seizure incidence within 24 h and rely on continuous EEG, whereas clinical detection (right) remains largely dependent on clinical observation, underestimating subclinical events. Central translational gaps include limited monitoring duration, use of young, healthy, male animal populations, and a poor understanding of how lesion volume and stroke severity relate to functional outcomes. Future directions emphasize standardization of continuous EEG across species, inclusion of aged and comorbid models, and systematic evaluation of prophylactic antiepileptic drug (AED) interventions. Figure created using Mind the Graph (https://mindthegraph.com).

Due to the cost and complexity of long-term studies, meaningful progress will require team grants which involve close collaboration between preclinical and clinical researchers. Such collaborations can ensure that animal models are designed with clinically relevant outcomes in mind, while clinical studies can, in turn, be informed by mechanistic insights derived from preclinical work. Dedicated funding streams are critical not only to sustain the extended timelines and resources required for long-term follow-up, but also to incentivize multidisciplinary partnerships that integrate expertise across neurology, neuroscience, pharmacology, and translational science. Without this coordinated effort, the translational gap between experimental findings and clinical practice is likely to persist. To visually summarize the key findings, translational gaps, and proposed directions bridging animal and human post-stroke seizure research, Figure 6 provides a schematic overview of major methodological differences, outcome patterns, and future priorities.

In conclusion, there is a critical need for targeted and methodologically rigorous preclinical PSS research. The high heterogeneity, significant risk of bias, and evidence of publication bias identified here call for urgent improvements in study design and reporting. By addressing these limitations, future preclinical studies can provide the robust, translatable evidence needed to inform clinical decision-making and ultimately improve the outcomes for the growing population of stroke survivors who face the debilitating consequences of PSE.

## Data Availability

The original contributions presented in the study are included in the article/Supplementary material, further inquiries can be directed to the corresponding author.

## Supplementary material

SUPPLEMENTARY FIGURE 1 Co-occurrence network of keywords using VOSviewer. This network visualization was generated from the 500 most common co-occurring keywords retrieved from a broad PubMed search of animal models of ischemia and hemorrhagic stroke. The analysis was conducted to informally evaluate the prevalence of preclinical PSS research. The resulting map highlights the absence of the terms "epilepsy" and "seizure," underscoring the scarcity of rigorous studies specifically investigating post-stroke seizures and post-stroke epilepsy in animal models.
