# Supplementary figures and images for: Post-stroke seizures in animal models: a systematic review and meta-analysis
Source: Front Neurosci. 2025 Dec 2;19:1716816. doi: 10.3389/fnins.2025.1716816 (PMC12705543; doi:10.3389/fnins.2025.1716816)

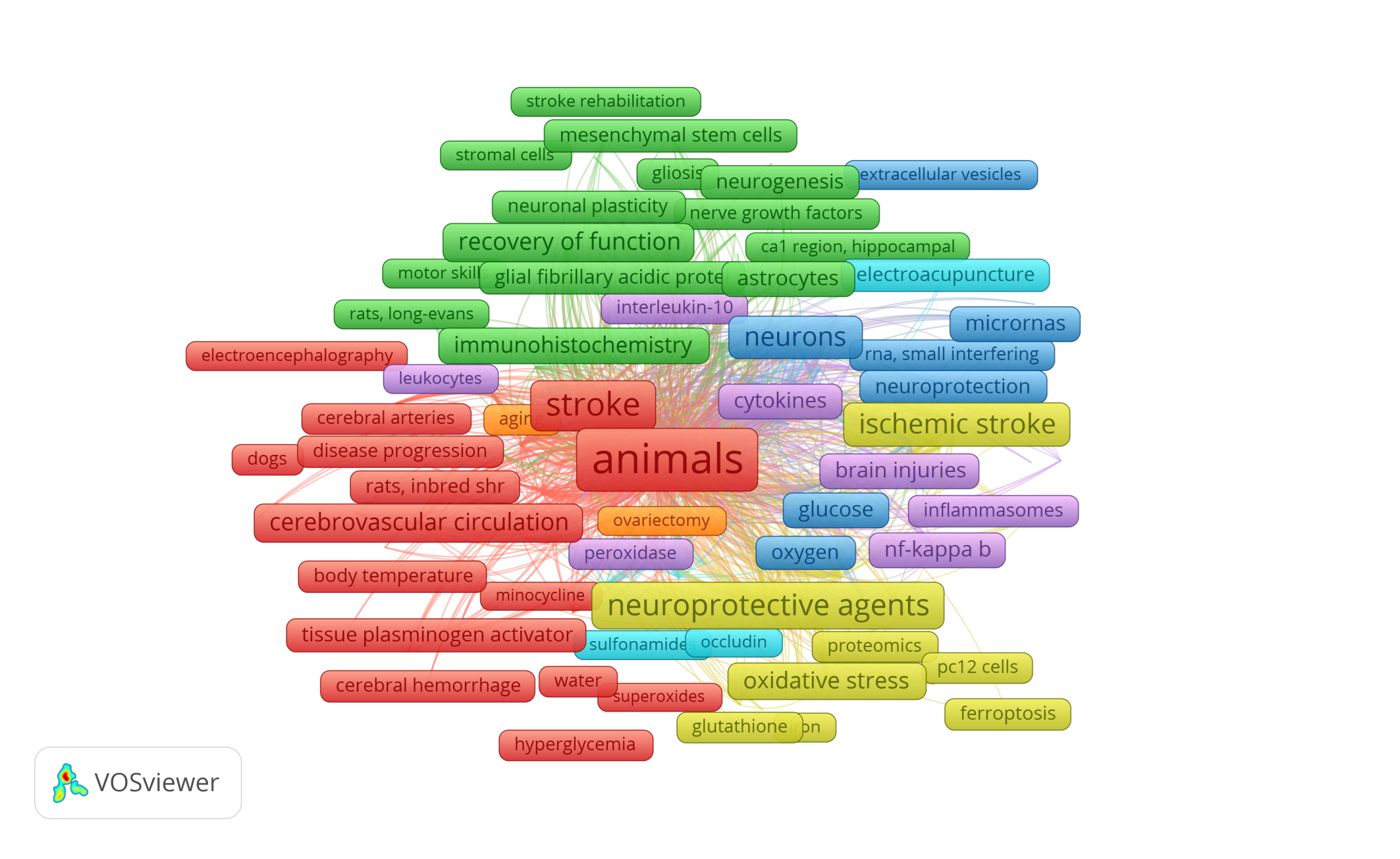

Supplement: SUPPLEMENTARY FIGURE 1 — Co-occurrence network of keywords using VOSviewer. This network visualization was generated from the 500 most common co-occurring keywords retrieved from a broad PubMed search of animal models of ischemia and hemorrhagic stroke. The analysis was conducted to informally evaluate the prevalence of preclinical PSS research. The resulting map highlights the absence of the terms "epilepsy" and "seizure," underscoring the scarcity of rigorous studies specifically investigating post-stroke seizures and post-stroke epilepsy in animal models. [file Image_1.TIF]
