# Supplementary material for: Post-stroke seizures in animal models: a systematic review and meta-analysis
Source: Front Neurosci. 2025 Dec 2;19:1716816. doi: 10.3389/fnins.2025.1716816 (PMC12705543; doi:10.3389/fnins.2025.1716816)
Supplement: Supplementary file 2 [file Data_Sheet_1.PDF]

Supplemental Table 1. Details and characteristics of studies included in the meta-analysis.

Study	Stroke Type	Age/ weight	Sex	Species, strain	Method to measure seizures	Seizure endpoints	Seizure Measurement Period	Main Endpoints	Euthanasia	% of Rats with Seizures
Germonpré et al., 2020	Collagenase ICH model	Age not stated; 250–350g	Male	Rats - Sprague Dawley	Continuous video-EEG (telemetry)	Number, number of animals with seizures, duration, laterality, latency, sleep/wake state prior to seizure, modified Racine score.	7 post-ICH	Lesion volume, cortical involvement, forelimb use asymmetry, sensorimotor deficits, NDS	Day 7	45.45%
Germonpré et al., 2021	Collagenase ICH model	Age not stated; 250–350g	Male	Rats - Sprague Dawley	Continuous video-EEG (telemetry)	Number, number of animals with seizures, duration, latency, modified Racine score	180 days post-ICH	Lesion volume, cortical involvement, ventricle dilatation, hemosiderin deposit volume, forelimb use asymmetry, spontaneous locomotor activity, inflammation markers	Day 180	50.00%
Hu et al., 2024	Embolic stroke model	2 month; 22–24g	Male	Mice - C57BL/6N	Intermittent video-EEG	Number of animals with seizures, RMS changes, behavioural signs of seizures	4–6 h post MCAO +10–14h	Lesion volume, rotarod test, NDS, inflammation markers	Day 2	50.00%
Jin et al., 2024	tMCAO (60 minutes) and tMCAO+CCAO	11–42 weeks; ~27g	Male and Female	Mice - C57BL/6J	Continuous video monitoring	Number of animals with seizures, latency	For 2 days after MCAO + CCAO	Lesion volume, mortality, edema, blood volume, NDS	Day 2	22.58% of the MCAO +CCAO animals
Karhunen et al., 2007	Photothrombotic model	9-12 weeks; 310-380g	Male	Rats - Sprague Dawley	Intermittent video-EEG (24h/day,7-14 days/session)	Number of animals with seizures, seizures per day, duration, and Racine score	2, 4-, 6-, 8- and 10-months post-stroke	Lesion depth and width, sensorimotor assessment, spatial memory, fear conditioning, hilar cell number, mossy fiber sprouting.	43-52 weeks	17.50%

Study	Stroke Type	Age/ weight	Sex	Species, strain	Method to measure seizures	Seizure endpoints	Seizure Measurement Period	Main Endpoints	Euthanasia	% of Rats with Seizures
Klahr et al., 2015	Collagenase ICH model and Whole blood ICH model	~3 month; 250–400g	Male	Rats - Sprague Dawley	Continuous EEG (telemetry)	Number, number of animals with seizures, laterality, duration, latency, RMS, coherence, power increases	7- and 30-days post-ICH	Lesion volume, temperature and activity	Day 7, 11–66 days	66.67% of the collagenase ICH model animals
Klahr et al., 2016	Collagenase ICH model	10–12 weeks; 350–450g	Male	Rats - Sprague Dawley	Continuous EEG (telemetry)	Number, number of animals with seizures, laterality, duration, latency, RMS, number of peaks during seizure	14 days post-ICH	Lesion volume, NDS, bleeding volume.	Day 14	59.09%
Lu et al., 2013	pMCAO	Age not stated; 275-350g	Male	Rats - Sprague Dawley	Continuous video-EEG	Number, number of animals with seizures, duration, latency, periodic epileptiform discharges, intermittent rhythmic delta activity	1-day post-pMCAO	Lesion volume	Not stated	82.14%
Tsai et al., 2011	Embolic stroke model	3 months old; 300-350g	Male	Rats - Wistar	Visual Observation	Percentage of animals with seizures	Not stated	Lesion volume, NDS, Cerebral blood flow	Not stated	39.9-66.04%
Wilkinson et al., 2020	Collagenase ICH model	3 month; 350–450g	Male	Rats - Sprague Dawley	Continuous EEG (telemetry)	Number, number of animals with seizures, laterality, duration, latency, RMS, coherence, power increases	2 days post-ICH	Lesion volume	Day 2	62.5%
